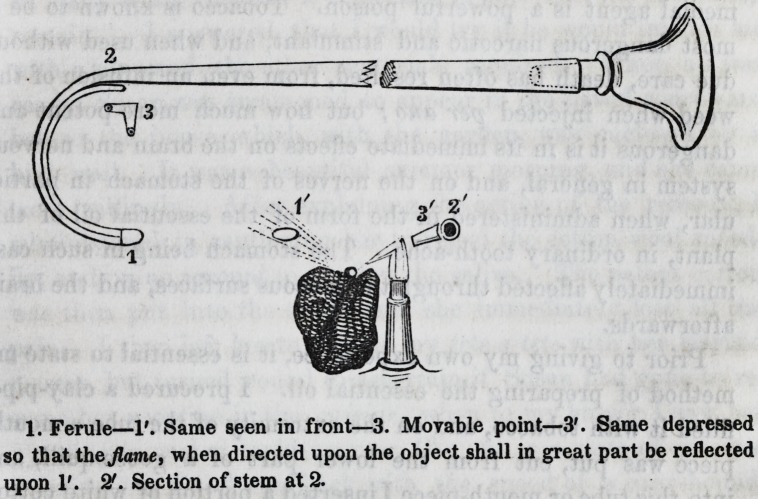# Hot Air Blow-Pipe

**Published:** 1854-01

**Authors:** Christopher Johnston


### ARTICLE VI.

*Hot Air Blow-pipe.* By CHRISTOPHER JOHNSTON, M. D., &c.

IT is hardly necessary to adduce arguments in favor of the *hot blast*, or its superiority over the *cold blast*, in the reduction of metals. But while the principle was acknowledged, its application was restricted to the grosser operations of manufacturers, and the experimental chemist, the analytical mineralogist combined to employ the ordinary blow-pipe in spite of its positive defects. To obviate the latter, I contrived a very simple instrument, had it constructed by Delevil, of Paris, and sent it in 1851, to my friend Dr. David Stewart.

The practical utility of the hot air blow-pipe has been established by that gentleman, and also by the Baltimore Academy of Arts and Sciences, before which I had the honor of exhibiting it.

The instrument consists of a tube of convenient length, tapering from the proximal to the distal end, and bent upon itself at the remote extremity so as to form a horse-shoe, of which the plane makes a small angle with the body tube, so that the middle of the curve shall not rest upon the charcoal used to support the object submitted to examination. The end of the tube now lies upon or against the body—upon it is fitted by friction a point bent at a right angle, and its proper direction may be attained by rotating the point upon the end of the tube.

An ivory mouth-piece is adapted by friction to the instrument, which gilding by the galvanic process, renders both cleaner and neater.

*Employment.*—The point (3) is adjusted in such manner that the *flame* impinging upon the metallic particle shall be reflected upon the knuckle or ferule (1) which becomes intensely heated, as does the AIR which passes through it. Moisture cannot interfere with the process, because upon reaching the heated ferule it is converted into surcharged steam, which, at the issuing point, escapes *above* the flame.

It is obvious that the knuckle or ferule cannot be melted, inasmuch as it loses caloric by radiation, and yields it to the air and the steam in process of *surcharging* within.

If the *point* be widely rotated upon its axis (longitudinal) the instrument may be used as a common blow-pipe.

**1. Ferule—1′. Same seen in front—3. Movable point—3′. Same depressed so that the *flame*, when directed upon the object shall in great part be reflected upon 1′. 2′. Section of stem at 2.**

## Figures and Tables

**Figure f1:**